# Weakened overturning and tide control the properties of Oyashio Intermediate Water, a key water mass in the North Pacific

**DOI:** 10.1038/s41598-021-93901-6

**Published:** 2021-07-15

**Authors:** Vigan Mensah, Kay. I. Ohshima

**Affiliations:** 1 Institute of Low Temperature Science, Hokkaido University, Sapporo, Japan; 2 Arctic Research Center, Hokkaido University, Sapporo, Japan

**Keywords:** Climate change, Ocean sciences

## Abstract

The western subarctic Pacific exhibits major biological productivity fed by the Oyashio Current and its two source waters: Western Subarctic Water, which supplies nutrients from the subarctic Pacific, and cold Okhotsk Sea Intermediate Water (OSIW), which supplies iron from the Sea of Okhotsk. We created seasonal climatologies of water properties to understand how the long-term trend (~ 50 years) and 18.6-year tidal cycle affect the Oyashio Intermediate Water (OYW). We found that over the trend, decreased OSIW outflow due to weakening of North Pacific overturning modifies OYW in winter. Meanwhile, OSIW outflow increases (decreases) in strong (weak) tide years. We predict that the opposite effects of the trend and strong tide will lead to stagnation of OYW properties until the mid-2020s, followed by accelerated warming until the mid-2030s (weak tide). A predicted 1 °C increase in OYW temperature and 50% decrease in OSIW content between 1960 and 2040 potentially have significant impact on biological productivity and carbon drawdown in the North Pacific.

## Introduction

The western subarctic Pacific possesses one of the largest biological CO_2_ drawdowns in the world^1,2^. A key contributor to this drawdown is the Oyashio, a western boundary current of the North Pacific Subarctic Gyre, which supplies the western subarctic with nutrients and iron^3^ via Oyashio Intermediate Water (OYW). OYW is a mixture of nutrient-rich Western Subarctic Gyre Water (WSAW) from the Bering Sea^4–7^ and cold, fresh, iron-rich Okhotsk Sea Intermediate Water (OSIW)^1,3,8^. In fact, OYW greatly contributes to the spreading of iron—an indispensable macronutrient for biological productivity—from the Sea of Okhotsk to the western subarctic region and the North Pacific Intermediate Water^9,10^. Meanwhile, the various nutrients contained in intermediate waters are redistributed towards the surface mainly via vertical turbulent flux near the Aleutians and the Kuril island chains^1^. This redistribution, together with the role played by OYW, controls the high biological productivity in the western subarctic Pacific^1^ and is thus influential to the major carbon drawdown and fisheries activities in the region.

Decades of global warming have greatly affected the Earth’s oceans. Several studies have traced the origin of isopycnal temperature changes in the northwestern Pacific to long-term warming trends in the Sea of Okhotsk^11–13^. The overturning of the northern Pacific waters also has been steadily weakening^12–14^. OSIW outflow into the Oyashio is an essential component of this overturning, in which dense water generated in winter at the surface of the Sea of Okhotsk sinks and mixes with surrounding waters to form OSIW before flowing out to the Pacific. Weakening of this overturning could reduce OSIW outflow. Bidecadal variability in the tides also could affect the northwestern Pacific^15–18^. Specifically, the mixing ratio of OSIW to OYW (i.e., the content of OSIW in OYW) seemingly increases during the strong period of the 18.6-year cycle of the diurnal tide and decreases during the weak part of the cycle^15,16^. This cycle thus also may affect OYW properties. Further quantification is needed to confirm the role and effects of the 18.6-year tidal cycle in the region and to compare these effects with those of long-term trend variability.

Many climate phenomena, such as the El Nino Southern Oscillation, are triggered by complex ocean–atmosphere interactions and occur irregularly^19–21^. Forecasting the effects of such phenomena is mostly achieved through modelling studies. By contrast, more predictable phenomena exist, such as the sinusoidal 18.6-year diurnal tide cycle. Besides, the long-term climate changes can be considered monotonous (linear-like) and are thus somewhat predictable too. As shown in this study, these two phenomena dominate the multi-decadal variability of OYW, implying that OYW properties can be predicted if the effects of the long-term trend and the tidal cycle are defined accurately for the region.

Proper quantification of these effects on the northwestern subarctic Pacific requires knowledge of the region’s seasonal variability. For example, large changes in the Oyashio region are expected to occur in winter when both the East Kamchatka Current, which transports WSAW, and the OSIW outflow into the northern Pacific are at their highest volumes^22–23^. However, studies in these regions often use yearly-averaged datasets^15,24^, which may be biased by the greater amount of data acquired in summer.

**Figure 1:**
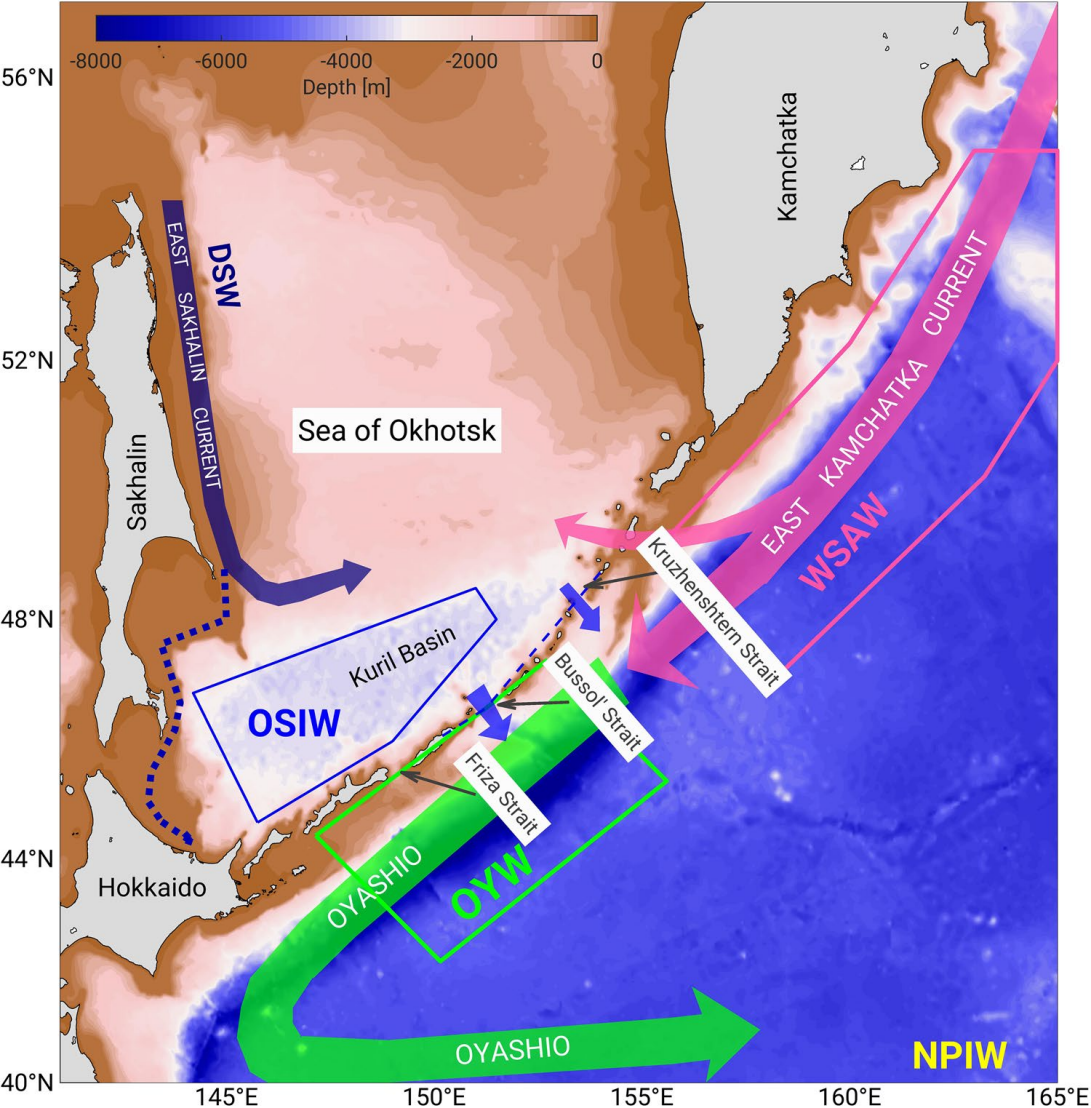
Schematic view of the study domain in the Sea of Okhotsk and the northwestern Pacific Ocean. The color shading represents the bathymetry. The pink, blue, and green boxes represent areas where the average properties of Western Subarctic Water, Okhotsk Sea Intermediate Water, and Oyashio Water are calculated, respectively. The dashed purple line represents a branch of the East Sakhalin Current flowing along the coast in winter. Dense shelf water (DSW) is produced on the northwestern shelf in the Sea of Okhotsk, and North Pacific Intermediate Water (NPIW) is formed partly from the Oyashio water (upper Oyashio is in the green box) and from intermediate water flowing with the Kuroshio further south (not shown).

Here, we established seasonal climatologies of temperature, salinity, and dissolved oxygen using historical data on the northwestern Pacific Ocean and Sea of Okhotsk (Fig. 1 and Supplementary Table S1). We used these climatologies to clarify the effects of the long-term trend and the 18.6-year tidal cycle in the Oyashio region. Based on these, we also provide a 20-year prediction of OYW properties. This study addressed seasonality, and we found that the greatest changes in Oyashio properties occur in winter. The substantial long-term decline in OSIW outflow to the northern Pacific, along with changes in the properties of OSIW and WSAW, have led to considerable warming of OYW over the past few decades. However, we also clarified that OSIW outflow increases during the strong period of the 18.6-year tide^15^, which is an opposite effect of the long-term trend.

Based on these observations, we predict that OYW properties will stabilize in the early 2020s as the 18.6-year tide strengthens. However, due to the tidal-induced decrease in OSIW outflow, enhanced warming and lower mixing ratios will occur from the mid-2020s to the mid-2030s when the 18.6-year tide is weak. We also predict that between the 1960s and 2030s, the OYW temperature will increase by 1 °C, and the mixing ratio will decrease by nearly 50%. These changes possibly limit the amount of iron in the northern Pacific, which might have major effects on the biological productivity and carbon uptake in this region.

## Results

### Seasonal effects and long-term changes in the northwestern Pacific

Temperature data from the study domain (Fig. 1) were mapped on isopycnal surfaces to create seasonal climatologies at each potential density (σ_θ_) layer in winter (January to April), summer (May to August), and fall (September to December) for the periods 1930–1990 and 1990–2020 (see “Methods” for details on the mapping methodology). The use of seasonal climatologies is reasonable as the residence time of the waters is relatively short in the East Kamchatka Current and Oyashio regions (within 1–2 months, assuming a current velocity of 0.1 m·s^−1^ to 0.2 m·s^−1^).

**Figure 2:**
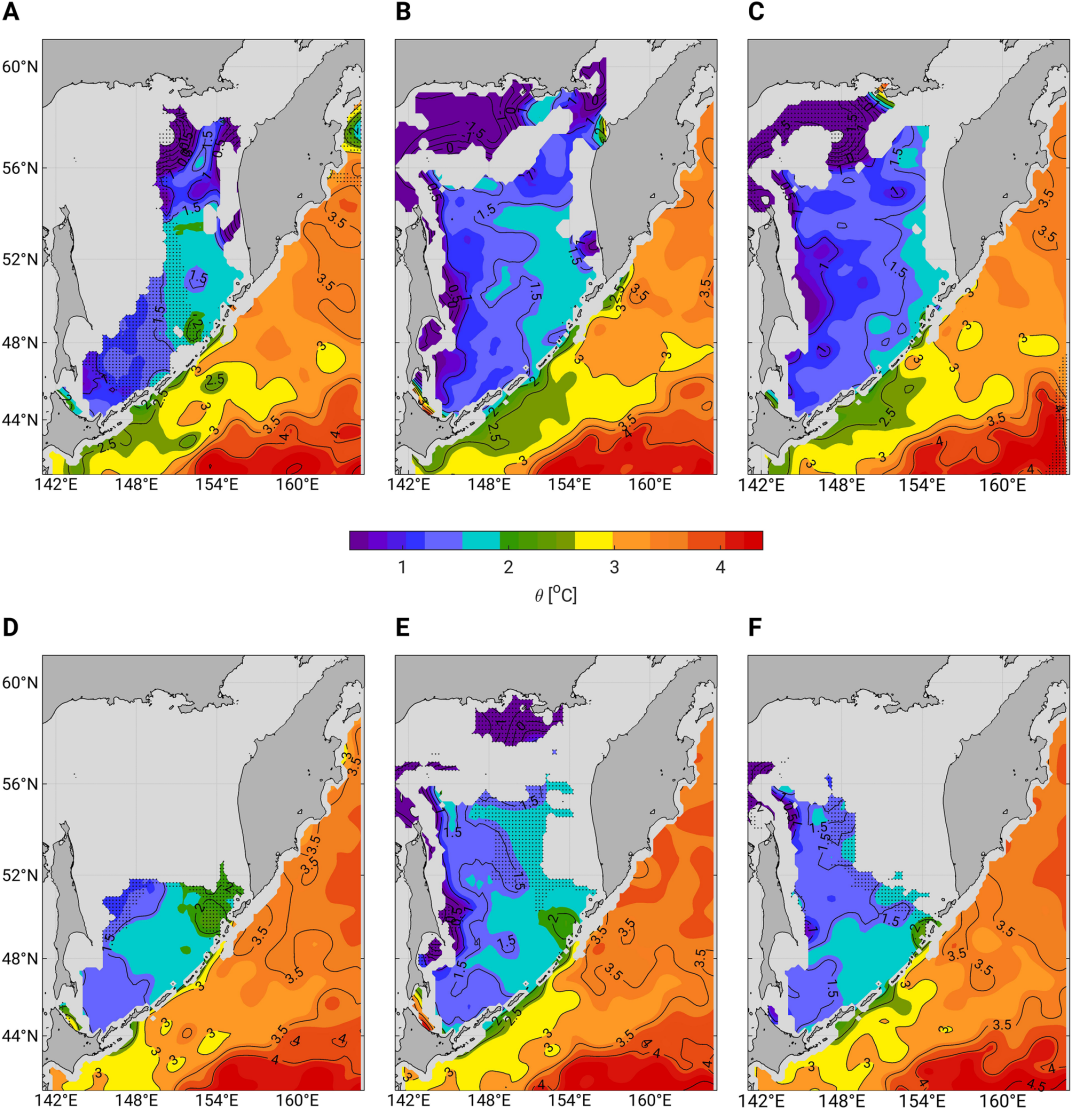
4-Month climatologies of potential temperatures in the Sea of Okhotsk and the northern Pacific at 26.9 σ_θ_. (**A**–**C**) 1930–1990 and (**D**–**F**) 1990–2020: (**A**,**D**) January to April, (**B**,**E**) May to August, and (**C**,**F**) September to December. The dashed areas represent data values calculated with 10–30 raw data points.

The climatologies from 1930–1990 at the 26.9 σ_θ_ layer (Fig. 2A–C) exemplify the basic properties of the domain’s water masses. Just outside of the Kuril Islands in the northern Pacific, water temperatures below 3 °C denote the presence of OYW. The rapid temperature decrease along the western boundary currents indicates the mixing of warmer WSAW (> 3.0 °C) from the north with colder OSIW (< 2.0 °C) from the Sea of Okhotsk^9,25^. Although OSIW outflow presumably increases in winter^22,23^, the area within the 3 °C isotherm is smaller in winter (Fig. 2A) than in summer and fall (Fig. 2B,C, respectively), likely due to the larger volume of warmer WSAW transported in winter with the East Kamchatka Current.

**Figure 3:**
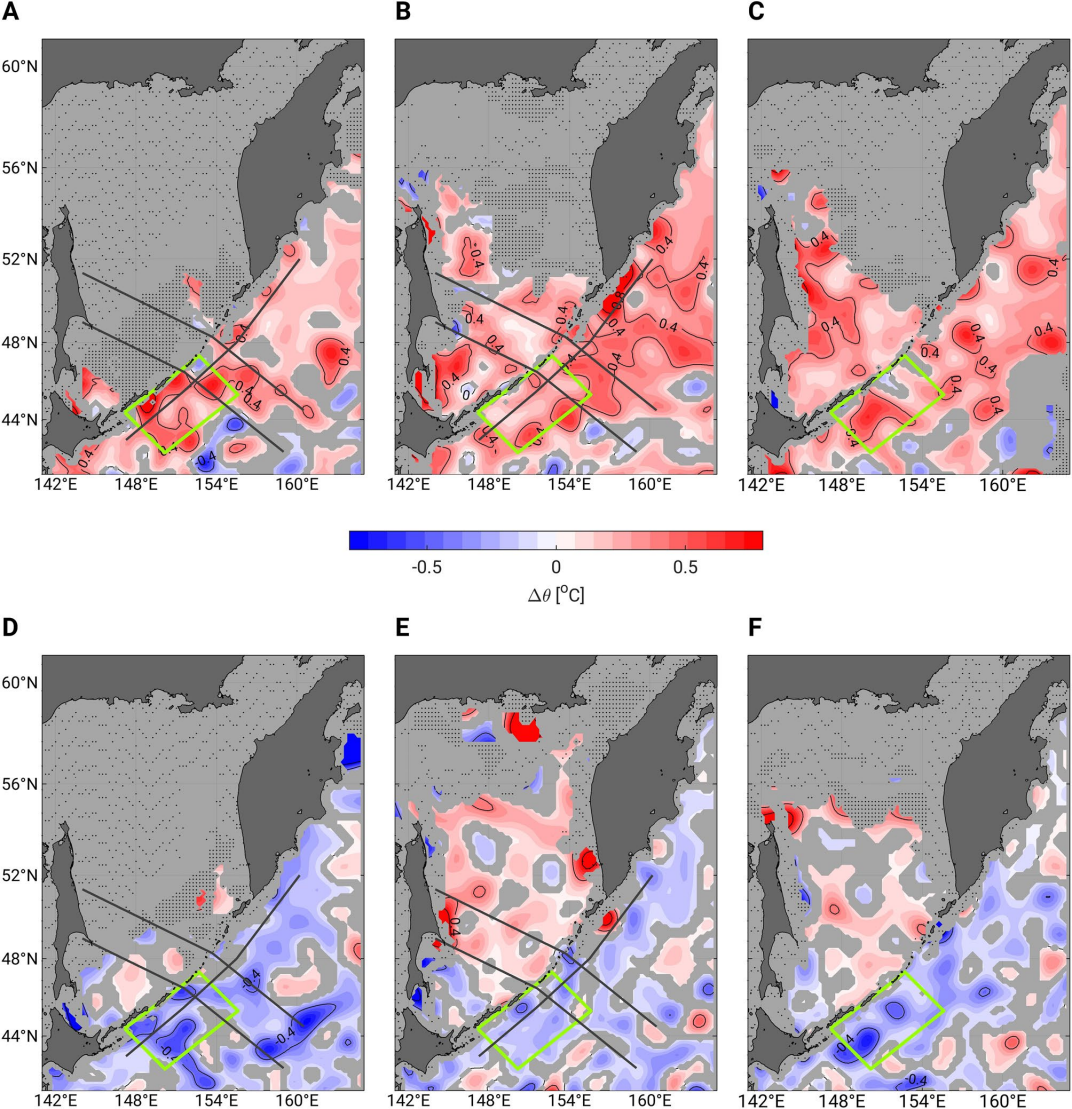
Maps of climatological differences in potential temperatures for the trend and the 18.6-year tidal cycle at 26.9 σ_θ_. (**A**–**C**) Difference between the 1990–2020 and the 1930–1990 climatologies, and (**D**–**F**) difference between the strong tide and the weak tide climatologies: (**A**,**D**) January–April, (**B**,**E**) May–August, (**C**,**F**) September–December. Areas with no data, or mapped with insufficient raw data (between 10 and 30 raw data points) remain uncolored and are filled with sparsely and densely distributed dots, respectively. Uncolored areas with no dot are regions where the climatological difference is smaller than the 95% confidence interval (see “Methods”). The three dark grey lines in (**A**,**B**,**D**,**E**) represent the three sections drawn in Fig. 4 and the green box represents the OYW definition area.

In the upper Oyashio region (the definition area for OYW, Fig. 1), the most striking differences between the climatologies for 1930–1990 and 1990–2020 occurred in winter (Fig. 3A). Temperatures in this area ranged between 2.5 °C and 3.0 °C from 1930 to 1990 (Fig. 2A), but they have increased by more than 0.4 °C since 1990 (Fig. 3A). Consequently, the OYW temperature mostly exceeded 3 °C from 1990 to 2020 (Fig. 2D). This contrast is less obvious in summer (Fig. 2B vs. 2E, Fig. 3B) and fall (Fig. 2C vs. 2F, Fig. 3C). In the Sea of Okhotsk, the 1990–2020 climatology indicates warmer temperatures in every season (Fig. 3A–C). This warming may be especially pronounced in the western part along the path of the East Sakhalin Current (Fig. 3B,C), which transports the dense shelf water produced on the northwestern shelf in winter. The cold and fresh signature of this water reaches the southern Sea of Okhotsk between May and August^26^. The higher summer (Figs. 2E, 3B) and fall (Figs. 2F, 3C) temperatures along the East Sakhalin Current in 1990–2020 thus may reflect intense winter warming of the dense shelf water over the past decades^13,14^.

**Figure 4:**
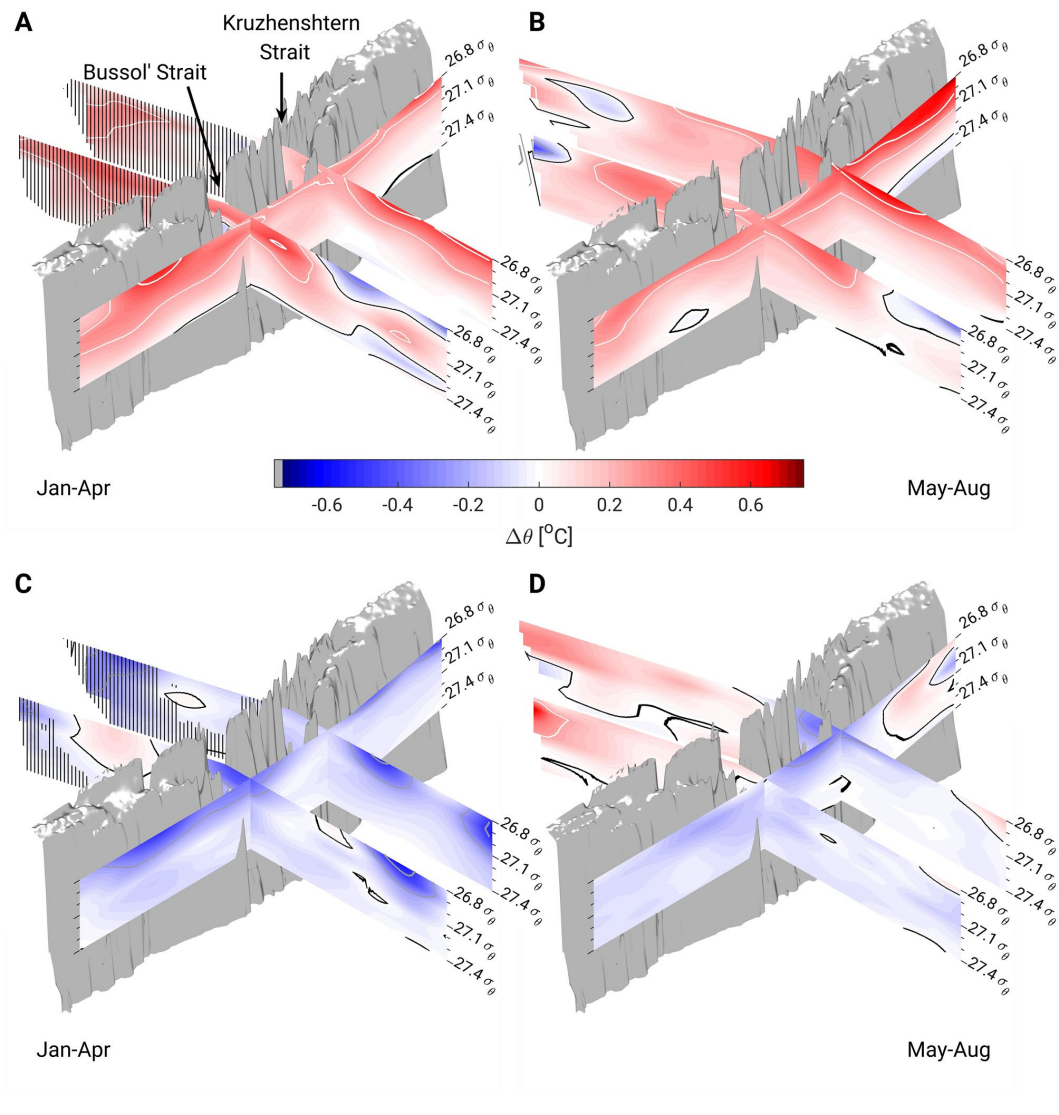
Cross-section of long-term trend and bidecadal (18.6-year tide) temperature anomalies on a σ_θ_-grid in the northern Pacific and the Sea of Okhotsk. From (**A**) January to April and (**B**) May to August. The anomalies every + 0.25 °C (− 0.25 °C) are represented by thin solid white (grey) lines and every + 0.5 °C (− 0.5 °C) by thick solid white (grey) lines. The thin solid black line represents the limit of the 0 °C anomaly. Barred vertical lines represent areas where values were calculated with 10–30 raw data points. (**C**,**D**) Same as (**A**,**B**) but for the 18.6-year tidal cycle, for which the temperature difference between strong tide years and weak tide years is calculated. To better reveal large-scale patterns, data along each section were smoothed with a 100-km half-width moving average filter. For non-smoothed data, the reader is referred to the map of temperature differences at 26.9 σ_θ_ in Fig. 3.

Smoothed temperature differences along the East Kamchatka Current–Oyashio path and across the Sea of Okhotsk also indicate intense warming in the upper Oyashio region in winter (Fig. 4A) and in the East Kamchatka Current region in summer (Fig. 4B). Along the section from Kamchatka to Hokkaido, the warm temperature anomaly increased in winter and decreased in summer just downstream of the Bussol' Strait, which has the largest outflow of OSIW^8,25,27^. The seasonal differences in the upper Oyashio region thus suggest that the decline in OSIW outflow to the northern Pacific is more prominent in winter than in other seasons.

### Opposite effects of strong tide versus long-term trend

The seasonal climatologies for weak and strong diurnal tide years also were estimated (Supplementary Fig. S1). The temperature difference between strong and weak tide years were estimated along three sections (Fig. 4C,D) and on the 26.9 σ_θ_ surface (Fig. 3D–F). The results suggest that the effects of the 18.6-year tide in strong years are opposite to those of the long-term trend (i.e., temperatures on the Pacific side of the Kuril Islands are lower during strong tide years than during weak tide years). Particularly large temperature differences occur in winter downstream of the Bussol' Strait (Figs. 3D, 4C), implying more prominent OSIW outflow during strong tide years. However, colder temperatures on the Pacific side of the Kuril Islands are unrelated to property changes in the Sea of Okhotsk, as the temperature differences in the Kuril Basin are either ill-defined (winter, Figs. 3D, 4C) or largely positive (summer and fall, Figs. 3E,F, 4D).

**Figure 5:**
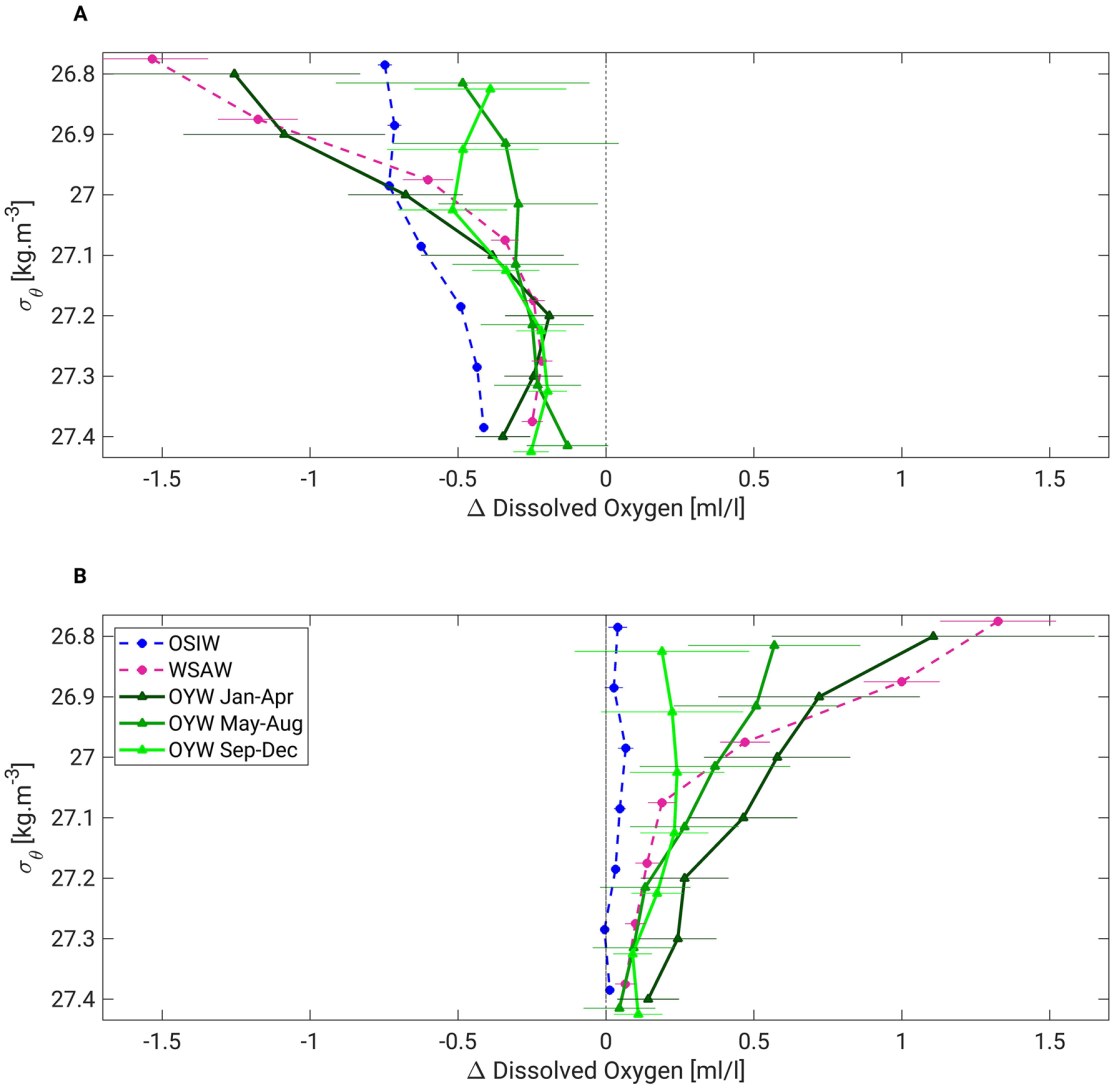
Dissolved oxygen changes due to long-term climate change and the 18.6-year tidal cycle. (**A**) Difference between the 1990–2020 and 1930–1990 climatologies, (**B**) difference between the strong and weak diurnal tide climatologies for OYW from January to April (dark green), May to August (medium green), and September to December (light green). To obtain the OSIW (blue dashed curves) and WSAW (pink dashed curves) values, all available DO data were used, regardless of the season. The error bars represent the 80% confidence interval (see “Methods” for details) for each curve. The data points and corresponding error bars are slightly shifted vertically for clarity.

The vertical profiles of dissolved oxygen (DO) show that between 1930–1990 and 1990–2020, the largest decrease in DO in the OYW occurred in winter (Fig. 5A). This decreasing trend is mirrored by an increase associated with a strong tide (Fig. 5B), that is also largest during winter. The long-term changes are similar in magnitude to those occurring between weak and strong tide years, both for DO (Fig. 5) and for temperature (Fig. 4). The results suggest that the strong 18.6-year tide tends to counter the effects of the long-term trend but that a weak diurnal tide exacerbates warming and DO decline caused by long-term climate change.

The long-term decrease in DO in OSIW (Fig. 5A) may be caused by weaker overturning associated with lower sea ice production over the past few decades^13–14^. Although causes for the DO decline in WSAW remain unclear, higher DO in strong tide years (Fig. 5B) could be related to intense tidal-induced mixing in the Aleutian Passes^18,28,29^. The results shown in Fig. 5 suggest that some changes in OYW properties relate to the variability of its source waters.

### Mixing ratio versus changes in source water properties

**Figure 6:**
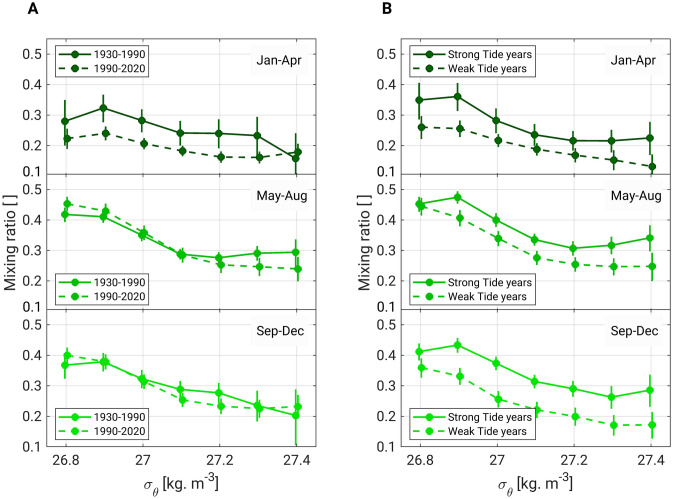
Seasonal mixing ratio in different climatological periods. (**A**) Mixing ratio averages for 1930–1990 and for 1990–2020 in winter (top), summer (middle), and fall (bottom). (**B**) Same as (**A**) for the mixing ratio averaged over strong and weak diurnal tide years. The error bars represent the 95% confidence interval (see “Methods” for details on the confidence interval and mixing ratio calculations). The data points and corresponding error bars are slightly shifted horizontally for clarity.

We calculated the mixing ratio of OSIW in OYW for every season to delineate the main causes of changes in temperature and DO content of OYW (Fig. 6). In the 26.8–27.0 σ_θ_ layers, the mixing ratio shows significant seasonal variability, from a minimum of 0.3 in winter to a maximum of 0.39 in summer (Fig. 6A, Supplementary Table S2). The largest changes between the 1930–1990 and 1990–2020 climatological periods occurred in winter (Fig. 6A), when the mixing ratio decreased by 28% in the upper intermediate waters (26.8–27.0 σ_θ_) and 23% in the lower intermediate waters (27.1–27.4 σ_θ_, Supplementary Table S2). Mixing ratio changes in summer and fall were insignificant (Supplementary Table S2). The large decrease in winter explains why we observed the strongest warming downstream of the Bussol' Strait in this season (Figs. 3A, 4A): the outflow of OSIW from the Sea of Okhotsk weakened over the long term, which led to a higher proportion of warm WSAW in OYW.

For the 18.6-year tidal cycle, the mixing ratio is higher in strong tide years than in weak tide years, regardless of the season (Fig. 6B). The magnitude of the tidal-related variations in the mixing ratio is comparable to that of the long-term trend in winter (Fig. 6A). This result further highlights the influence of the tidal cycle on OYW properties. The higher mixing ratio in every season during strong tide years demonstrates higher OSIW outflow into the northern Pacific, as proposed in Ref.^15^. This increased outflow enhances cooling (Figs. 3D–F, 4C,D) and increases the DO content (Fig. 5B) of OYW. The largest increases in mixing ratio occur in winter in the upper intermediate waters (+ 38%, Supplementary Table S2) and in fall in the lower intermediate waters (+ 53%).

**Figure 7:**
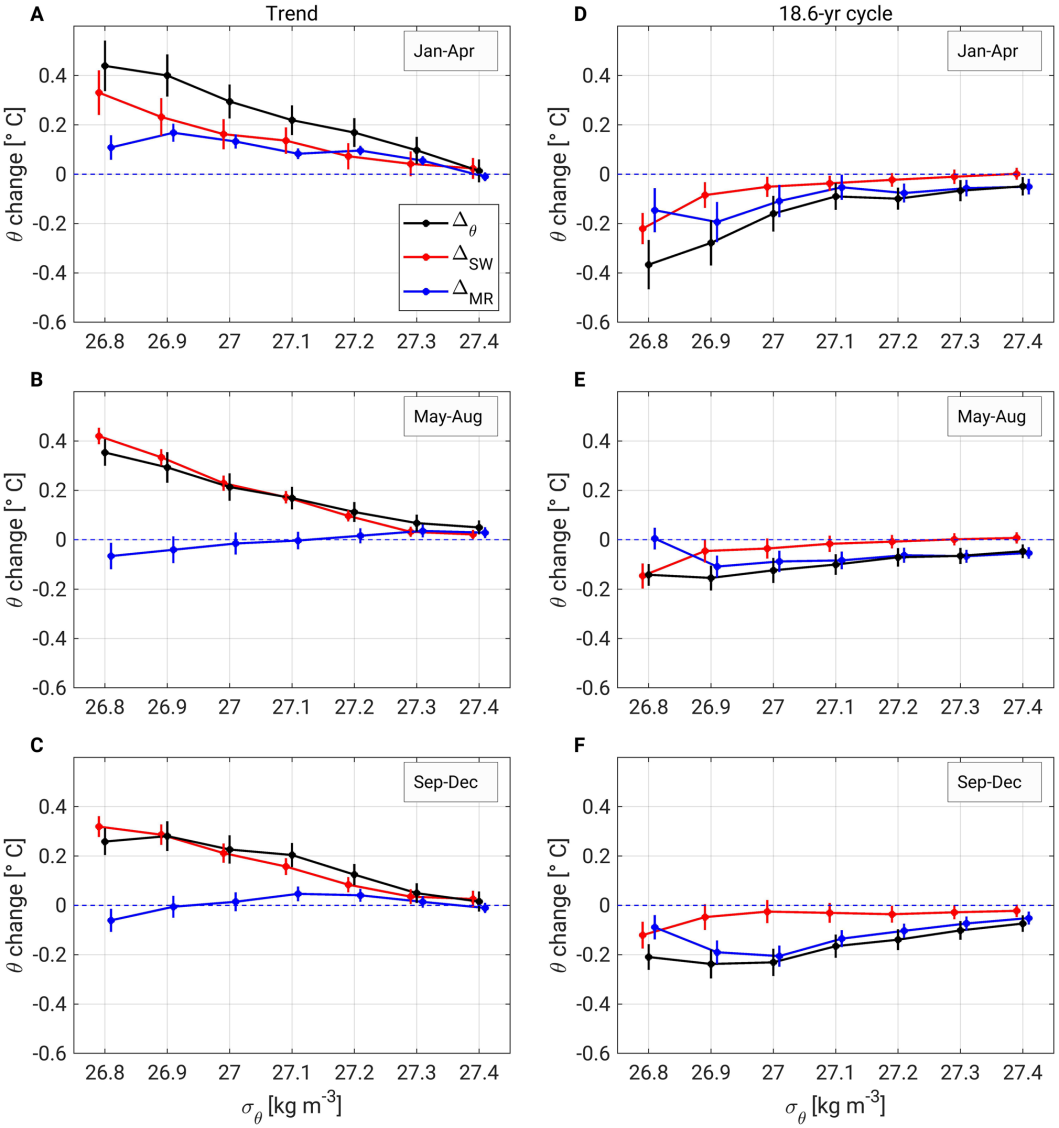
Potential temperature changes in OYW and their contributing factors. Changes in OYW potential temperature (black) and the contribution to these variations by changes in the mixing ratio (Δ_MR_, blue) and changes in the source water properties (Δ_SW_, red) from (**A**,**D**) January to April, (**B**,**E**) May to August, and (**C**,**F**) September to December. Panels (**A**–**C**) represent the changes due to the long-term trend and (**D**–**F**) the changes due to the 18.6-year tidal cycle. The error bars represent the 95% confidence interval (see “Methods” for details on the confidence interval calculation). The data points and corresponding error bars are slightly shifted horizontally for clarity.

We also quantified the temperature changes in OYW and how much of these changes were caused by variations in the mixing ratio Δ_MR_ (blue curves in Fig. 7) and by changes in the source water (OSIW and WSAW) temperatures Δ_SW_ (red curves in Fig. 7). Long-term warming of OYW (Fig. 7A–C) peaked in winter seasons (0.40 °C at 26.9 σ_θ_, 0.22 °C at 27.1 σ_θ_), and slightly less than half of this warming was caused by changes in the mixing ratio (0.17 °C at 26.9 σ_θ_, 0.08 °C at 27.1 σ_θ_). The warming of the source waters can be considered a direct effect of global warming on OYW temperature, whereas warming due to the decreased mixing ratio is an indirect effect of global warming caused by a dynamical mechanism: the reduction of OSIW outflow into the northern Pacific Ocean due to weakened North Pacific overturning (see “Discussion”). These two contributions (Δ_SW_ and Δ_MR_) were equally large only in winter, which explains OYW’s most extreme warming in this season (Figs. 3A, 4A) and possibly its loss of DO content (Fig. 5A). In contrast, most OYW warming in summer and fall (~ 0.28 °C at 26.9 σ_θ_, ~ 0.20 °C at 27.1 σ_θ_) was due to warming of the source waters (i.e., OYW properties were mostly affected directly by global warming during these seasons).

The results for the 18.6-year tidal cycle (Fig. 7D–F) show that the change in mixing ratio is the main contributor to OYW temperature variations in every season, except at 26.8 σ_θ_. We suggest that a dynamical mechanism also leads to variation in OSIW outflow and governs changes in OYW properties over the 18.6-year tidal period^15^ (see “Discussion”). In particular, we adopt the concept delineated in Ref.^30^ that the tidal current is essential for water exchange between the Sea of Okhotsk and the northern Pacific. As the intensity of the tidal current should increase during the strong period of the 18.6-year tidal cycle, so should the outflow of OSIW into the northern Pacific.

Finally, it should be noted that the interpretation of our results assumes isopycnal mixing even though strong, tidal-related diapycnal mixing occurs around the Kuril Islands^27–29^. This assumption could limit the validity of our conclusions. However, the effects of diapycnal mixing on water properties in the western subarctic Pacific are minor^31^ and likely confined to the regions around these islands. Furthermore, the results of climatologies mapped by excluding all data within 50 km of the Kuril Islands, where diapycnal diffusivity is high^31^ (not shown), are not significantly different from those delineated in this paper. Our conclusions should therefore not be negatively affected by our assumption of isopycnal mixing.

### Future predictions of Oyashio water properties

**Figure 8:**
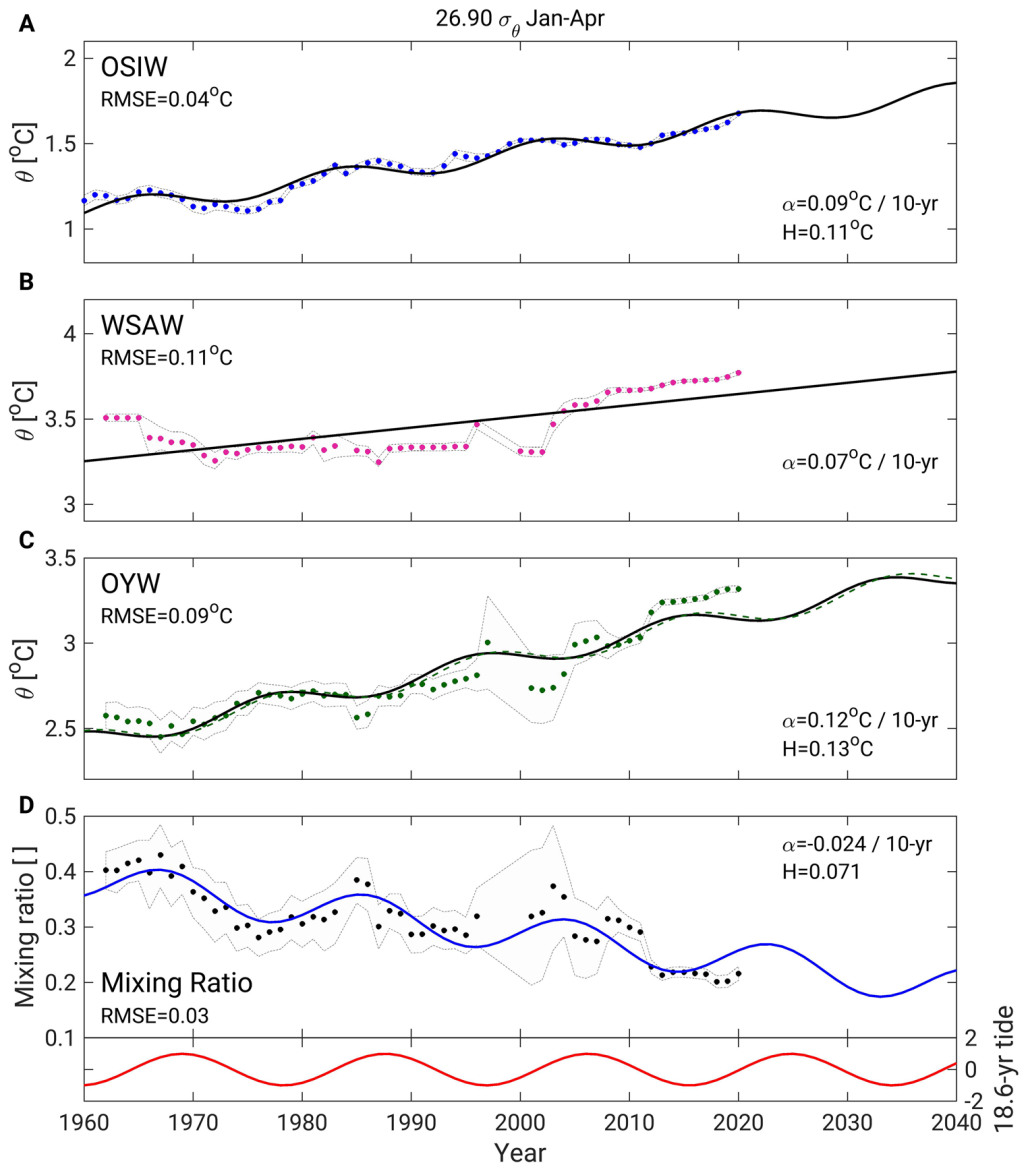
Time series of observed and predicted winter properties in the Sea of Okhotsk and western subarctic Pacific at 26.9 σ_θ_. Dots represent (mapped) observations, and lines predict data for (**A**) yearly-averaged OSIW, (**B**) January–April WSAW temperatures, (**C**) January–April OYW temperatures, and (**D**) January–April mixing ratio. In (**C**), the thin green dashed line represents predicted OYW temperatures estimated from Eq. (1). The red curve in the lower panel in (**D**) represents the 18.6-year diurnal tidal cycle. In each panel, the root mean square error (RMSE) indicates the root mean square error between observations and predictions, α indicates the linear 10-year trend, and H indicates twice the amplitude of the tidal signal (i.e., the difference over a half 18.6-year tidal period). In each panel, the light gray shading represents the 95% confidence interval (see “Methods” for details on the confidence interval calculation).

The long-term climate change effects on OYW can be considered monotonous and those of the 18.6-year tide cyclic, allowing us to make predictions about OYW properties. After establishing yearly time series of winter data from 1960 to 2020 for OSIW, WSAW, and OYW temperatures, as well as mixing ratios (see “Methods”), we fit a linear model to the WSAW data and a linear-sinusoidal model^15^ to the OSIW and mixing ratio data as follows (see “Methods” for details about our choice of fitting model):

1 $$ \Gamma  = O + A \cdot (y - y_{0} ) + h \cdot \cos (2\pi (y - y_{0}  - \varphi )/18.6), $$

where *y* is year (*y* = 1960, 1961, …, 2020), y_0_ = 1969 is a reference year for the tide^15^ (peak strong tide year), and the offset *O*, trend *A*, sinusoid amplitude *h*, and phase lag *φ* are set to minimize the sum of the squared residuals between the prediction model and the data. Once the models are established, *y* was extended to 2040 to obtain predictions of WSAW (*T*_p_WSAW_) temperature, OSIW temperature (*T*_p_OSIW_), and mixing ratio (MR_p_). The predicted curves for OSIW temperature and for the mixing ratio fit the data well, with root mean square errors of 0.04 °C and 0.03, respectively (Fig. 8A,D). These good fits highlight how the 18.6-year tidal oscillations modulate the increasing OSIW temperature and decreasing mixing ratio trends. The WSAW temperature time series showed no significant 18.6-year oscillation (Fig. 8B), thus a linear model was fitted to these data instead (see details in “Methods”). We then predicted OYW temperature based on the assumption that OYW results from the isopycnal mixing of OSIW and WSAW^8,13,15,24,25^ as follows:

2 $$ T_{{{\text{p\_OYW}}}}  = MR_{{\text{p}}}  \cdot T_{{{\text{p\_OSIW}}}}  + (1 - MR_{{\text{p}}} ) \cdot T_{{{\text{p\_WSAW}}}} . $$

The prediction models for 26.9 σ_θ_ yielded warming trends of 0.12 °C∙10-year^−1^, 0.07 °C∙10-year^−1^, and 0.09 °C∙10-year^−1^, respectively, for OYW, WSAW, and OSIW (Fig. 8A–C). We thus predict that between 1960 and 2040, the OYW temperature will increase by 1.0 °C due to the long-term climate trend. As demonstrated in the previous section, the changes in temperature of the two source waters of OYW explain only about half of the total increase in OYW temperature. The remaining warming is due to the descending mixing ratio (Fig. 8D). With a change of − 0.024∙10-year^−1^ in this ratio, the content of OSIW in OYW would decrease by 0.19 between 1960 and 2040, which represents half the value estimated for the 1960s (0.39).

The amplitude of the 18.6-year periodic signal for OYW (0.13 °C, Fig. 8C) yielded by our prediction model is smaller than that estimated from the climatological comparison for 26.9 σ_θ_ (0.30 °C, Fig. 7D), and the same is true of the mixing ratio (0.071 in Fig. 8D versus 0.10 in Fig. 6B). Less data were available for the period 1997–2008, which led to larger 95% confidence intervals and higher dispersion of data points around the prediction model (Fig. 8C,D) during this period. These factors, along with smoothing due to our yearly mapping scheme (see “Methods”) and variability of periods shorter than a decade, likely blurred the 18.6-year tidal signal in the time series and led to its underestimation in our model. The relatively large error bars of the winter climatological data in Figs. 6B and 7D also may explain the discrepancy between these data and the time series.

## Discussion

Our analysis revealed a strong seasonality in the long-term temperature variations in the upper Oyashio region. The temperature changes were highest in winter, mainly due to variability of the OSIW outflow into the northern Pacific. The mixing ratio of OSIW in OYW decreased by nearly 30% during winters over the long-term, leading to considerable warming and decreased DO content of OYW. We consider two different dynamical mechanisms for this decrease in OSIW content in the Oyashio: a change in the Sverdrup transport and weakening of the overturning. We consider the validity of these two hypotheses below.

A main driver of the exchange of water between the northern Pacific and the Sea of Okhotsk is the wind stress curl, either via the Sverdrup transport averaged between the latitudes of the northernmost and southernmost Kuril Straits^23^ or via the East Kamchatka Current volume transport at the northernmost strait^32^. If the winter wind stress curl declines, the exchange system between the northern Pacific and Sea of Okhotsk also weakens and thus OSIW outflow and mixing ratio decrease. However, both the East Kamchatka Current volume transport and the winter Sverdrup transport, calculated following Ref.^23^ between 1958 and 2019, have increased by about 10% of the typical 20–30 Sv (1 Sv = 10^6^ m^3^∙s^−1^) East Kamchatka Current transport^22,23,33^, which rules out this possibility.

We propose instead that weakened overturning of the Pacific is the main cause for the decrease in the proportion of OSIW in OYW. The Okhotsk Sea is the only location in the northern Pacific where dense water forms and sinks to the intermediate level^34,35^, and thus it is an essential location for overturning. From 1960 to 2000, the production of dense shelf water has reportedly decreased by about 30% at 26.8–27.1 σ_θ_^13^, which is similar to the drop in the mixing ratio observed over a similar period (Fig. 6A). Specifically, the following chain of events may explain the loss in mixing ratio. First, atmospheric warming over the past decades in the northern Sea of Okhotsk^36^ decreased sea ice and dense shelf water productions^13,14^, thus weakening the overturning cell in winter. This weakening then caused a reduction in intermediate water outflow from the Sea of Okhotsk to the northern Pacific, hence the reduced mixing ratio. With less cold OSIW reaching the upper Oyashio region, OYW warmed and its DO content decreased substantially. Thus, global warming may have contributed significantly to changes in OYW properties, both by warming OYW’s source waters, and by weakening the North Pacific overturning in winter (Supplementary Figs. S6). Sea ice and DSW are typically no longer produced at the end of winter, which may explain why the effects of the overturning weakening are less pronounced in summer and fall. The rapid transmission (within one season) of the overturning weakening from the Sea of Okhotsk’s northwestern shelves to the Kuril Straits implies that the transmission mechanism is a linear dynamical response rather than an advective process. Determining the nature of such transmission mechanism could be the subject of future works.

The average outflow of OSIW for layers between 26.8–27.0 σ_θ_ has been estimated to be around 1.4 Sv^24^. A value of 2.2 Sv was estimated from synoptic survey data in the range of 26.7–27.0 σ_θ_^25^. The seasonal variability of OSIW outflow is suggested to be strong^23^, but it has not been evaluated yet. Therefore, quantification of the seasonal variability of OSIW outflow is needed.

A comparative analysis revealed that OYW changes induced by the 18.6-year tidal cycle were similar in magnitude to those related to the long-term trend. Variations in OSIW outflow is the main factor governing the 18.6-year variability of OYW properties. Reference^15^ offers two possible mechanisms to explain this variation. First, OSIW outflow changes might be due to increases in OSIW layer thickness during strong tide years and decreases during weak tide years. The time series of OSIW temperatures (Fig. 8A) and layer thickness (Supplementary Fig. S5C) at 26.9 σ_θ_ show oscillations in the 18.6-year period. However, these oscillations lag the 18.6-year tide (Fig. 8D) by 4 years. In contrast, the 18.6-year periodic variations of mixing ratio precede those of the 18.6-year tide by 2 years (Fig. 8D). We assumed that the mixing ratio and tidal cycles are quasi-simultaneous and that the 2-year difference is due only to the relative lack of available data in winter (Supplementary Table S1). The composite of three seasons of mixing ratio time series yielded only a 1-year difference between the mixing ratio and tidal cycles (Supplementary Fig. S5A), which supports our assumption. The 5–6 years of lag in the maximum layer thickness to the maximum mixing ratio means that thickness variations cannot explain changes in OSIW outflow. Instead, we suggest that the tidal current influences the exchange system between the Sea of Okhotsk and the northern Pacific and leads to variations in OSIW outflow^15^, as explained in a recent modelling study^30^. The 4-year lag in layer thickness to the tidal cycle may relate to how OSIW properties are remotely affected by slow advection of surface salinity anomalies from the Bering Sea to the Sea of Okhotsk^15,37,38^. The exact interpretation for this 4-year lag is left for future investigations.

By accurately describing the effects of the long-term trend and the tidal cycle, we made several predictions about the properties of OYW. Specifically, between 1960 and 2040, OYW temperatures at 26.9 σ_θ_ will increase by about 1 °C, and the mixing ratio will decrease by nearly 50%. Until the mid-2020s, the effects of the long-term trend and of the strong 18.6-year tide will counteract each other, and the temperature and mixing ratio will likely stagnate. However, from the mid-2020s to the mid-2030s, the weak tide will further decrease OSIW outflow and exacerbate the effects of long-term warming. These effects will lead to a predicted winter mixing ratio as low as 0.18 in 2034, compared to a local maximum of 0.44 in 1967. This decrease in OSIW content could lead to decreases in iron and DO in Oyashio waters. Changes in OYW properties should ultimately affect the characteristics of North Pacific Intermediate Water. Specifically, because biological productivity is limited by iron availability, reduced OSIW outflow could lead to loss of productivity and weakening of the carbon drawdown in the subpolar and subtropical northern Pacific. The quantification and prediction of these changes are essential issues that merit further investigations.

## Methods

### Data sources

The hydrographic data used in this study originate from the World Ocean Database 2018^39^, supplemented by data acquired by the Russian research vessel Khromov between 1998 and 2007. Additional data included conductivity, temperature, and depth measurements from 29 floats deployed as part of a joint study conducted by Japan, Russia, and the United States and a cooperative study between Hokkaido University and the University of Washington^13,23^. Float data were acquired from 2000 to 2019, and 9 of the floats acquired oxygen data which were corrected for drift and bias, following previous research^40^. Four-month (January–April, May–August, September–December) climatological maps were then computed for 1930–1990 and 1990–2020 and used to evaluate the effects of the long-term trend. The median year is 1977 for the 1930–1990 winter dataset and 2014 for the 1990–2020 winter dataset, for a difference of 37 years between the two climatologies. The periods for strong and weak tides were determined using previous methods^15^. Weak tide periods occurred from September 1919 to December 1928, April 1938 to July 1947, December 1956 to March 1966, July 1975 to October 1984, February 1994 to May 2003, and September 2012 onward. Strong tide periods correspond to the interval years. Supplementary Table S1 shows the temperature/salinity and DO raw data available in the study region for each climatological period. Climatologies were established for each period via “ensemble mapping” (see the Ensemble mapping subsection). The long-term trend anomaly was obtained by calculating the difference between the 1990–2020 and 1930–1990 climatologies. The effects of the 18.6-year tidal cycle were determined by calculating the difference between the strong tide and weak tide climatologies.

### Ensemble mapping

We used 4-month climatology periods as a trade-off between a time span short enough to represent seasonal changes and long enough to include sufficient data for the analysis. Climatologies were calculated on isopycnal surfaces in the density range of 26.8–27.4 σ_θ_ for temperature, salinity, and DO. The climatologies were estimated by objectively mapping raw data onto a 1/6°Lat × 1/4°Lon horizontal grid, following Ref.^41^, with the e-folding radius set to 150 km based on Refs.^13,24^. Grid-cell values estimated from fewer than 10 data points were excluded from the results. Mapping was conducted separately for the Sea of Okhotsk and the North Pacific to avoid cross-basin smoothing.

The different climatological periods included more data from strong (weak) tide years in the 1930–1990 (1990–2020) period (Supplementary Table S3). This uneven data distribution could lead to overestimation of the temperature trends, because Oyashio water is warmer (colder) during weak (strong) tide years. To mitigate this issue, the climatologies of temperature and salinity were obtained via ensemble mapping. Ensemble mapping averages *n* (*n* = 10) mapped climatologies, each obtained from a subset of the data listed in Supplementary Table S1. For each climatological period, the size of the subset was determined by the number of data points acquired within the least sampled tidal cycle. For example, in winter in the North Pacific, 609 (882) data points were acquired during weak (strong) tide years during 1930–1990 (Supplementary Table S3). The subset thus included 609 data points from weak tide years and 609 randomly selected data points acquired during strong tide years. One climatological map was obtained from these data, and the operation was repeated *n* times with different random data selections. All our analyses and figures were based on the average of the *n* maps. At each grid point, the ensemble standard deviation offered a measure of the mapping repeatability. This process also was used to estimate the confidence intervals (see the Water mass properties subsection).

Ensemble mapping also was used to establish climatologies for the strong and weak tide years. In this case, all data were first detrended based on smoothed versions of the trend maps in Fig. 3A–C. Then, for each climatological period, subsets including 75% of the total number of data points were used to produce *n* maps, which were subsequently averaged. Ensemble mapping was used for temperature and salinity, but the DO dataset was too small; thus, DO climatologies were established from all available data.

### Water mass properties

The isopycnal potential temperature, salinity, DO, and layer thickness of OYW, WSAW, and OSIW for each climatological period (Figs. 5, 7, and 8 and Supplementary Figs. S2–S5) were determined by averaging the mapped data with mapping errors^41^ of less than 0.05 within the areas delineated in Fig. 1. The properties of OSIW used for calculating the mixing ratio (explained in the next subsection) and illustrated in Figs. 5, 8 and Supplementary Figs. S2–S5, were calculated from all data available, regardless of the season. Indeed, because the residence time of OSIW in the Sea of Okhotsk is greater than 1 year^23,42–44^, we did not expect the properties of outflowing OSIW to have a seasonal signature. The 95% (80%) confidence intervals for temperature (DO) were estimated for each density layer (σ) and water mass at each season as follows:

3a $$ CI(\sigma ,\;t) = 1.96~(1.24) \cdot ~\left( {\Sigma (\sigma ,\;t)/\sqrt {N_{d} }  + \overline{{\Gamma (\sigma ,\;t)}} /\sqrt n  + ~\overline{{ME(\sigma ,\;t)}} } \right), $$

where Σ is the standard deviation of all the mapped data within a water mass definition area (colored boxes in Fig. 1), *N*_*d*_ is the number of raw data used in the objective mapping within the definition areas (i.e., the number of independent points within each respective area), *t* is the season (*t* = 1 for January–April, *t* = 2 for May–August, and *t* = 3 for September–December), Γ is the ensemble standard deviation (see the Ensemble mapping subsection), *n* is the number of maps used to create an ensemble (*n* = 10), and the overbar indicates spatial averaging over the water mass definition areas. ME is the absolute mapping error obtained using Ref.^45^ as follows:

3b $$ \overline{{ME}} (\sigma ,\;t) = \frac{1}{N}\int_{{i = 1}}^{{i = N}} {\varepsilon (\sigma ,\;t,\;i) \times \delta (\sigma ,\;t,\;i)} . $$

ε is the mapping error from Ref.^41^ expressed as a percentage of the variance in grid cell *i* (*i* = 1,…, *N,* where N is the number of grid cells within the waters’ respective definition areas), and δ is a grid-cell standard deviation of all raw data located within one e-folding radius (150 km) of the center of grid cell *i*. For DO differences in the long-term trend (18.6-year) in Fig. 5A (Fig. 5B), an 80% confidence interval for each water mass was obtained by adding the 80% confidence interval of 1930–1990 (weak tide years) to that of 1990–2020 (strong tide years). Due to the lower number of DO data points available in winter in the WSAW region, the DO values for WSAW also were estimated from all the data available, regardless of season.

For the maps of climatological differences of Fig. 3, the confidence interval at each grid point in position (*x*, *y*) and season was established as follows:

4a $$ CI_{{map}} (x,\;y) = ~CI_{1} (x,\;y) + CI_{2} (x,\;y), $$

4b $$ CI_{{1,~2}} (x,\;y) = 1.96~ \cdot ~\left( {\Gamma (x,\;y)/\sqrt n  + ~ME(x,\;y)} \right) $$

where *CI*_1_ and *CI*_2_ represent the confidence interval for a given climatological period (e.g., 1930–1990 for *CI*_1_, 1990–2020 for *CI*_2_). Γ and ME are the same quantities as described in Eqs. (3a, 3b), without spatial averaging. Temperature differences below the confidence interval are not shown in Fig. 3.

### Mixing ratio estimates and relative contributions to temperature changes

We calculated the mixing ratio from the θ-S properties of OSIW, WSAW, and OYW at each density level (Supplementary Fig. S2) for each season by assuming that OYW results from along-isopycnal mixing of OSIW and WSAW, as assumed in numerous previous works^8,13,15,24,25^. Based on this, the mixing ratio MR_θ_ at each density layer for each season was calculated as follows:

5 $$ MR_{\theta } (\sigma ,\;t) = \frac{{~\theta _{{WSAW}} (\sigma ,\;t)~ - ~\theta _{{OYW}} (\sigma ,\;t)~}}{{{{\theta _{{WSAW}} (\sigma ,\;t) - ~\theta }} _{{OSIW}} (\sigma )~}},~ $$

where *θ*_*OYW*_ and *θ*_*WSAW*_ are the potential temperatures of OYW and WSAW, respectively, at a given density level σ (26.7, 26.8 … 27.5) and season *t*. $\bar{\theta }_{{OSIW}}$ is the yearly average potential temperature of OSIW at density level σ. The relationship linking potential temperature *θ* and salinity *S* to potential density σ_*θ*_ is not linear. Thus, mixing ratio MR_S_, based on salinity data, was also calculated similarly to Eq. (5). Supplementary Table S2 lists the MR values, which were obtained by averaging MR_θ_ and MR_S_. The 95% confidence interval of the values in Supplementary Table S2 were obtained by recalculating MR and replacing each *θ* term in Eq. (5) with *θ* ± *CI*(*σ,t*), with CI defined as in Eq. (3a).

To calculate the contribution of the change in mixing ratio of OSIW to the OYW temperature variations over the long-term trend, we first estimated the properties that a synthetic OYW would have had in 1990–2020 if it were composed of the θ_*WSAW*_ and $\bar{\theta }_{{OSIW}}$ of 1990–2020 but with the mixing ratio of 1930–1990:

6 $$ \theta _{{OYW_{{syn}} }} (\sigma ,\;t) = \bar{\theta }_{{OSIW_{{9020}} }} (\sigma ) + (\bar{\theta }_{{OSIW_{{9020}} }} (\sigma ) - ~\theta _{{WSAW_{{9020}} }} (\sigma ,\;t))~ \times (1 - MR_{{\theta \_3090}} (\sigma ,\;t)). $$

The contribution of the mixing ratio to the temperature changes was then obtained by subtracting the synthetic OYW potential temperature from the actual 1990–2020 OYW potential temperature.

7 $$ \Delta \theta _{{MR}} (\sigma ,\;t) = \theta _{{OYW\_9020}} (\sigma ,\;t) - \theta _{{OYW\_syn}} (\sigma ,\;t). $$

Last, considering that the total change in OYW potential temperature is:

8a $$ \Delta \theta (\sigma ,\;t) = \theta _{{OYW\_9020}} (\sigma ,\;t) - \theta _{{OYW\_3090}} (\sigma ,\;t), $$

the contribution of the source water properties change to Δθ is simply

8b $$ \Delta \theta _{{SW}} (\sigma ,\;t) = \Delta \theta (\sigma ,\;t) - \Delta \theta _{{MR}} (\sigma ,\;t). $$

Alternatively, the contribution by the source waters can be expressed using Eqs. (7), (8a), and (8b) as follows:

8c $$ \Delta \theta _{{SW}} (\sigma ,\;t) = \theta _{{OYW\_syn}} (\sigma ,\;t) - \theta _{{OYW\_3090}} (\sigma ,\;t). $$

The same method was used to calculate these properties for the 18.6-year tidal cycle. The confidence intervals for Δθ_MR_, Δθ, and Δθ_SW_ were obtained by replacing each *θ*(*σ,t*) term in Eqs. (7), (8a), and (8c) by its respective confidence interval CI(*σ,t*) and replacing the – sign with a + sign.

### Time series and prediction model

The time series displayed in Fig. 8 and Supplementary Figs. S3–S5 were obtained by averaging objectively mapped temperatures within the respective areas of OSIW, WSAW, and OYW (Fig. 1). For each season, climatologies were established separately for each year from 1960 to 2020. Each climatology at year *y* was estimated by mapping all data within *y* ± 4 years, without ensemble mapping. The 95% confidence intervals were obtained following Eq. (3a), and the mixing ratio was obtained for each year following Eq. (5). Once the time series were obtained, the data were least-square fitted to either a first-degree linear model or to the linear-sinusoidal model described in Ref.^15^ and Eq. (1). To determine which of the two models was the most adequate to represent a given time series (i.e., to ensure that the model did not overfit the data), we used the corrected Akaike Information Criterium^46^:

9 $$ AICc~ = ~n~\times~\log (SSE{\text{/}}n)~ + ~(n~ + ~p)~/~(1~ - ~(p~ + ~2)~/~n), $$

where *n* is the number of data points, *SSE* is the sum of squared residuals between the model and data, and *p* is the number of fitting parameters (*p* = 2 for the linear model and *p* = 4 for the linear-sinusoidal model). The corrected Akaike Information Criterium was calculated for both models, and the model with the smallest criterium was considered the most appropriate to describe the data. The criteria of the linear-sinusoidal model were smaller for OSIW temperature (− 327 versus − 288 for the linear model), thickness time series (253 versus 309), and mixing ratio time series (− 313 versus − 286). The criterium of the first-degree linear model was smaller only for the WSAW temperature time series (− 180 versus − 179 for the linear-sinusoidal model).

## Supplementary Information

Supplementary Information.

## Data Availability

The datasets generated and analyzed during the current study are available from the corresponding author on reasonable request.
