# Supplementary material for: Weakened overturning and tide control the properties of Oyashio Intermediate Water, a key water mass in the North Pacific
Source: Sci Rep. 2021 Jul 15;11:14526. doi: 10.1038/s41598-021-93901-6 (PMC8282869; doi:10.1038/s41598-021-93901-6)
Supplement: Supplementary file 1 — Supplementary Information. [file 41598_2021_93901_MOESM1_ESM.docx]

Supplementary Materials for

**Weakened overturning and tide control the properties of Oyashio Intermediate Water, a key water mass in the North Pacific**

Vigan MENSAH*, Kay. I. OHSHIMA

*Corresponding author. Email:[vmensah@lowtem.hokudai.ac.jp](mailto:vmensah@lowtem.hokudai.ac.jp)

**This PDF file includes:**

Figs. S1 to S6

Tables S1 to S3


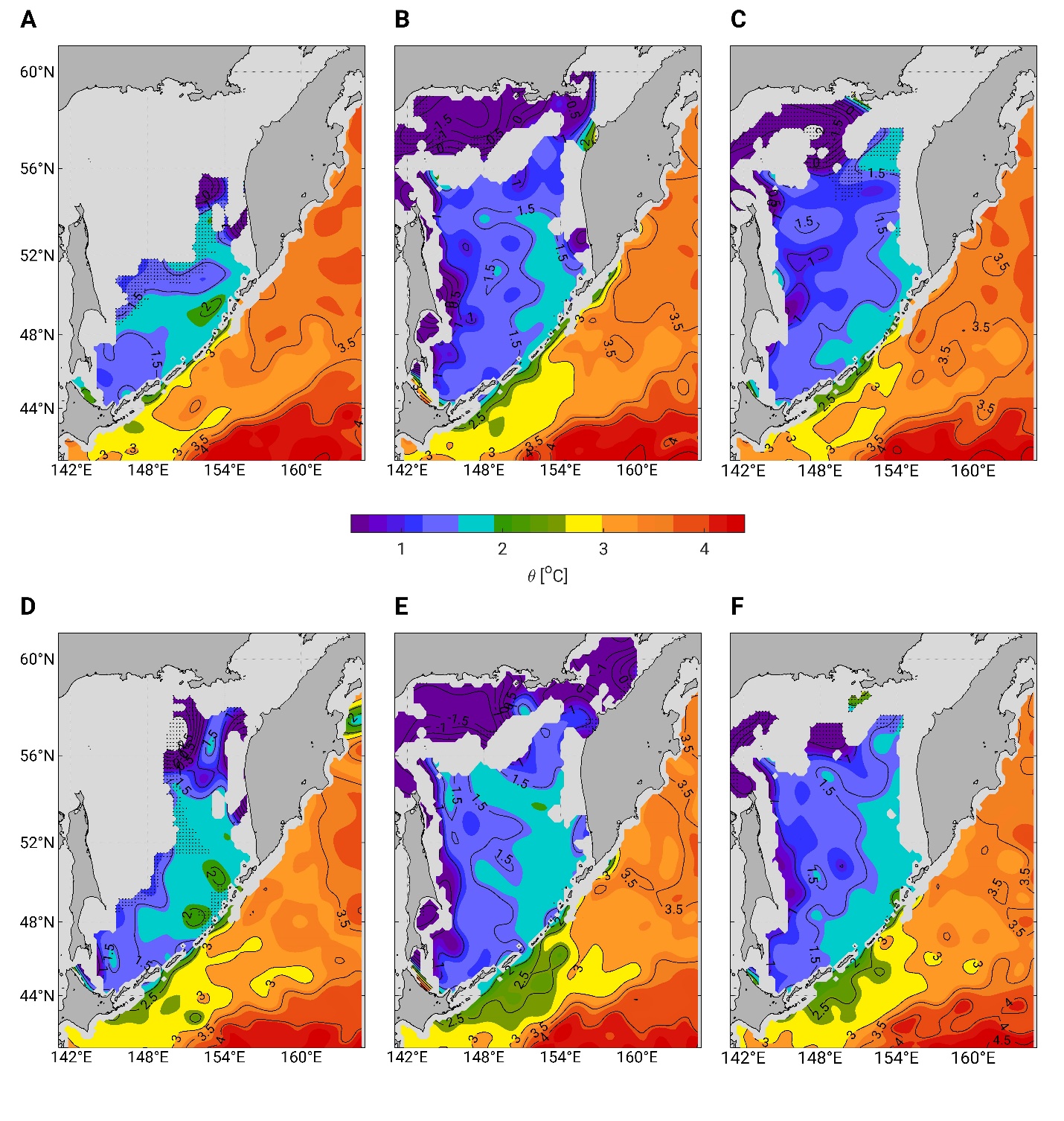


Fig. S1. 4-month climatologies of potential temperatures in the Sea of Okhotsk and the western subartic Pacific at 26.9 σ_θ._ (A-C) Low-tide years, and (D-F) high-tide years: (A, D) January-April, (B, E) May-August, (C, F) September-December. The dashed areas represent data values calculated with between 10 and 30 raw data points. The figure was drawn using MATLAB version R19a (The MathWorks, Inc. <https://uk.mathworks.com/products/matlab.html>) and the m_map toolbox developed by R. Pawlowicz, and available online [www.eoas.ubc.ca/~rich/map.html](https://www.eoas.ubc.ca/~rich/map.html).


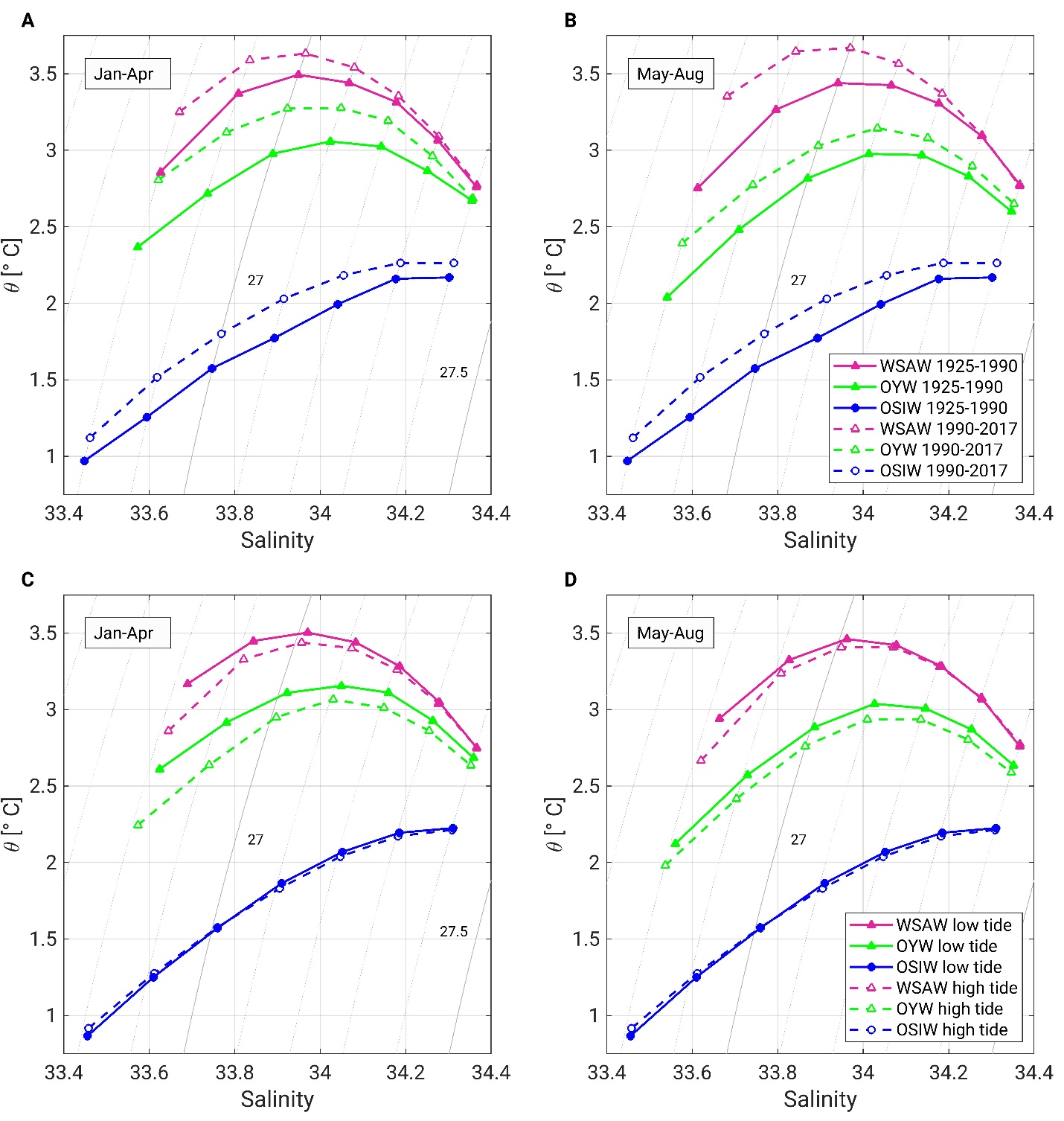


Fig. S2. θ-S diagrams of water masses in the Sea of Okhotsk and the western subarctic Pacific. OYW (green), WSAW (pink) and OSIW (blue) in (A, C) January-April, and (B, D) May-August for the climatological periods representative of the long-term trend (A, B) and the 18.6-yr tidal cycle (C, D).


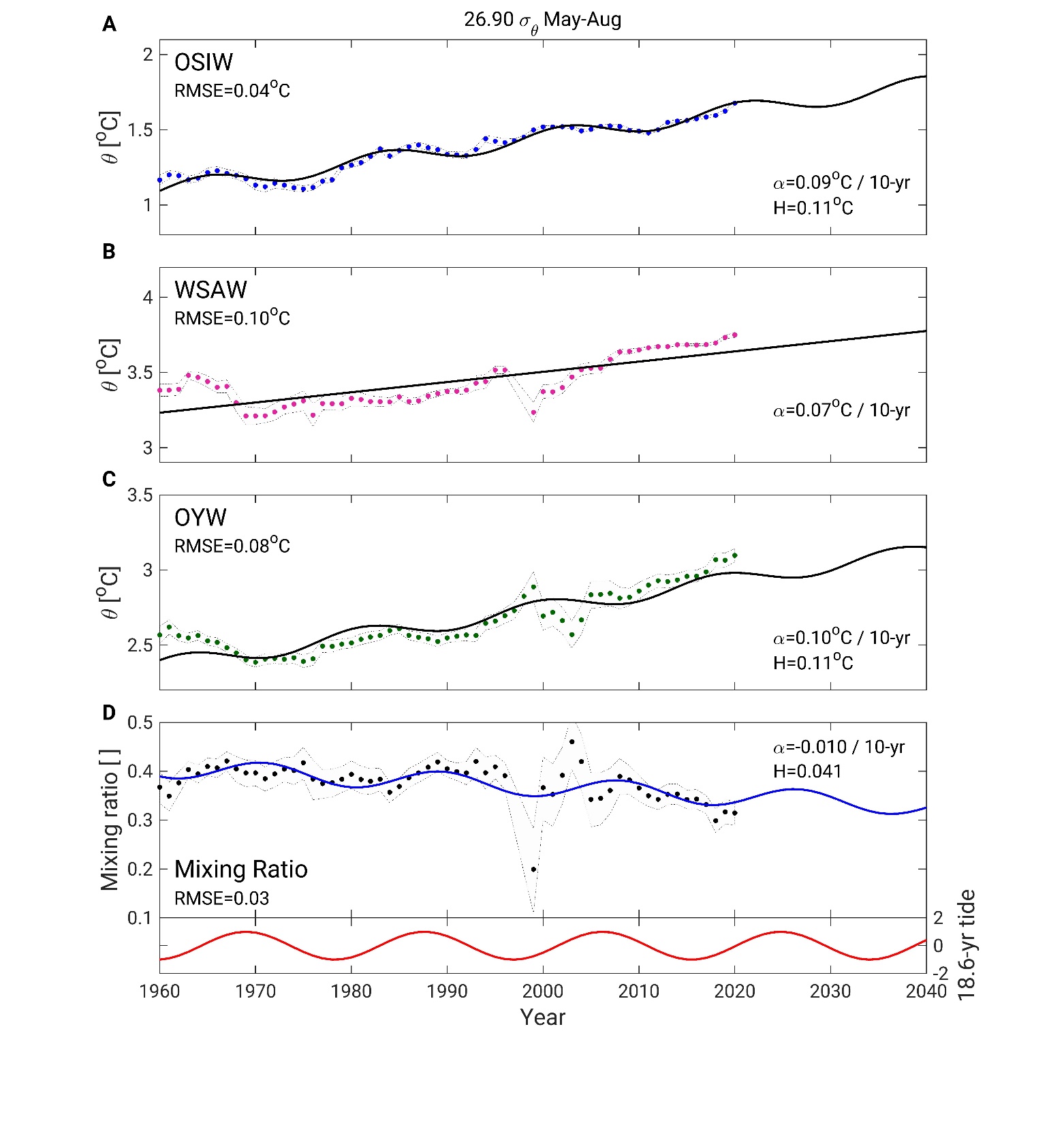


Fig. S3. Time series of observed and predicted summer properties in the Sea of Okhotsk and western subarctic Pacific at 26.9 σ_θ_. Dots represents (mapped) observations and line predicted data for (A) yearly-averaged OSIW, and (B) May-Aug WSAW temperatures, (C) May-Aug OYW temperatures, (D) May-Aug mixing ratio. The red curve in the lower panel in (D) represents the 18.6- year diurnal tidal cycle. In each panel, the RMSE indicates the root mean square error between observations and predictions, α indicates the linear 10-year trend, and H indicates twice the amplitude of the tidal signal (i.e., the difference over a half 18.6-year tidal period). In each panel, the light gray shading represents the 95% confidence interval.


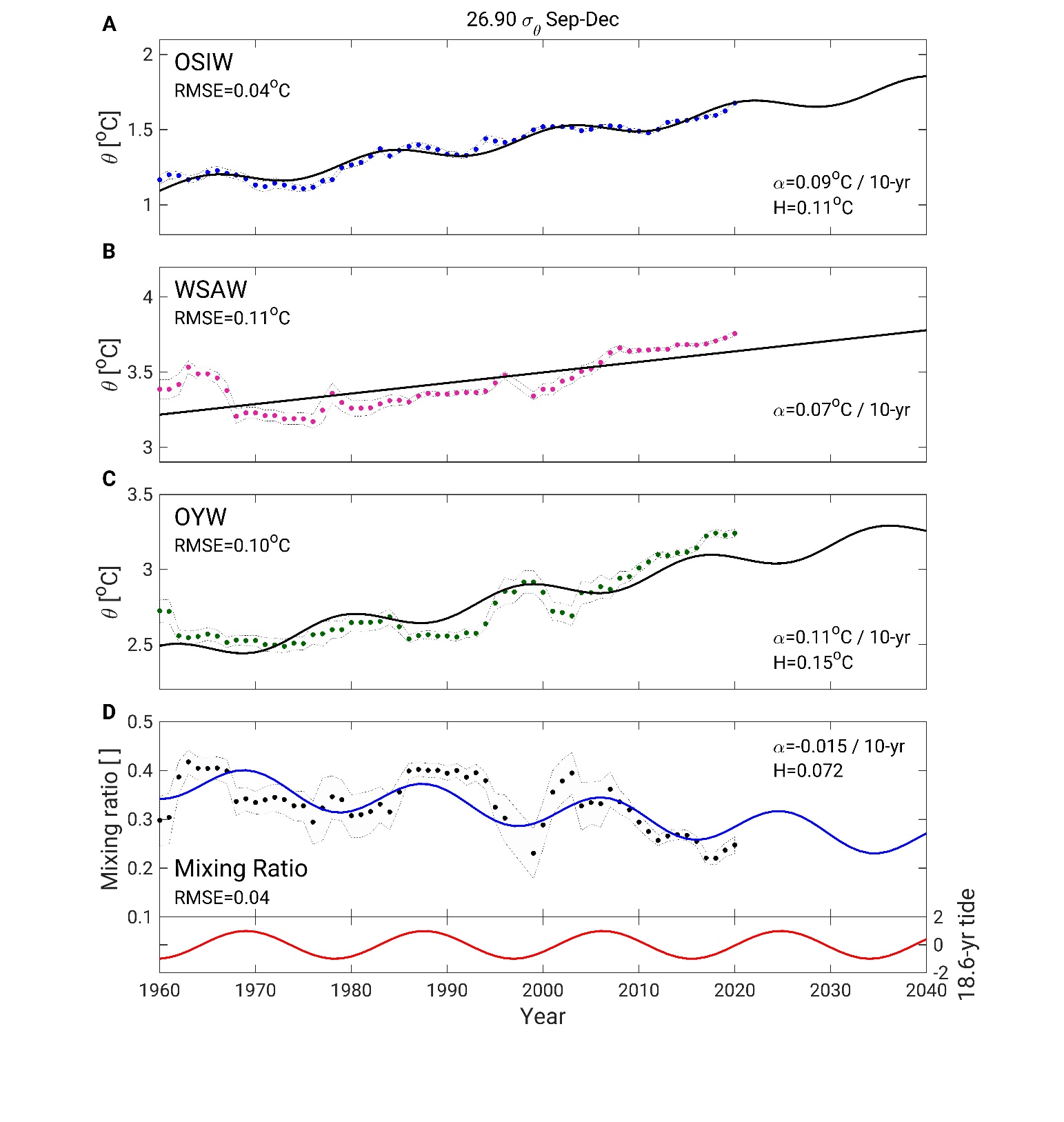


Fig. S4. Time series of observed and predicted fall properties in the Sea of Okhotsk and western subarctic Pacific at 26.9 σ_θ_. The line types and colors are identical to those of Fig. S3.


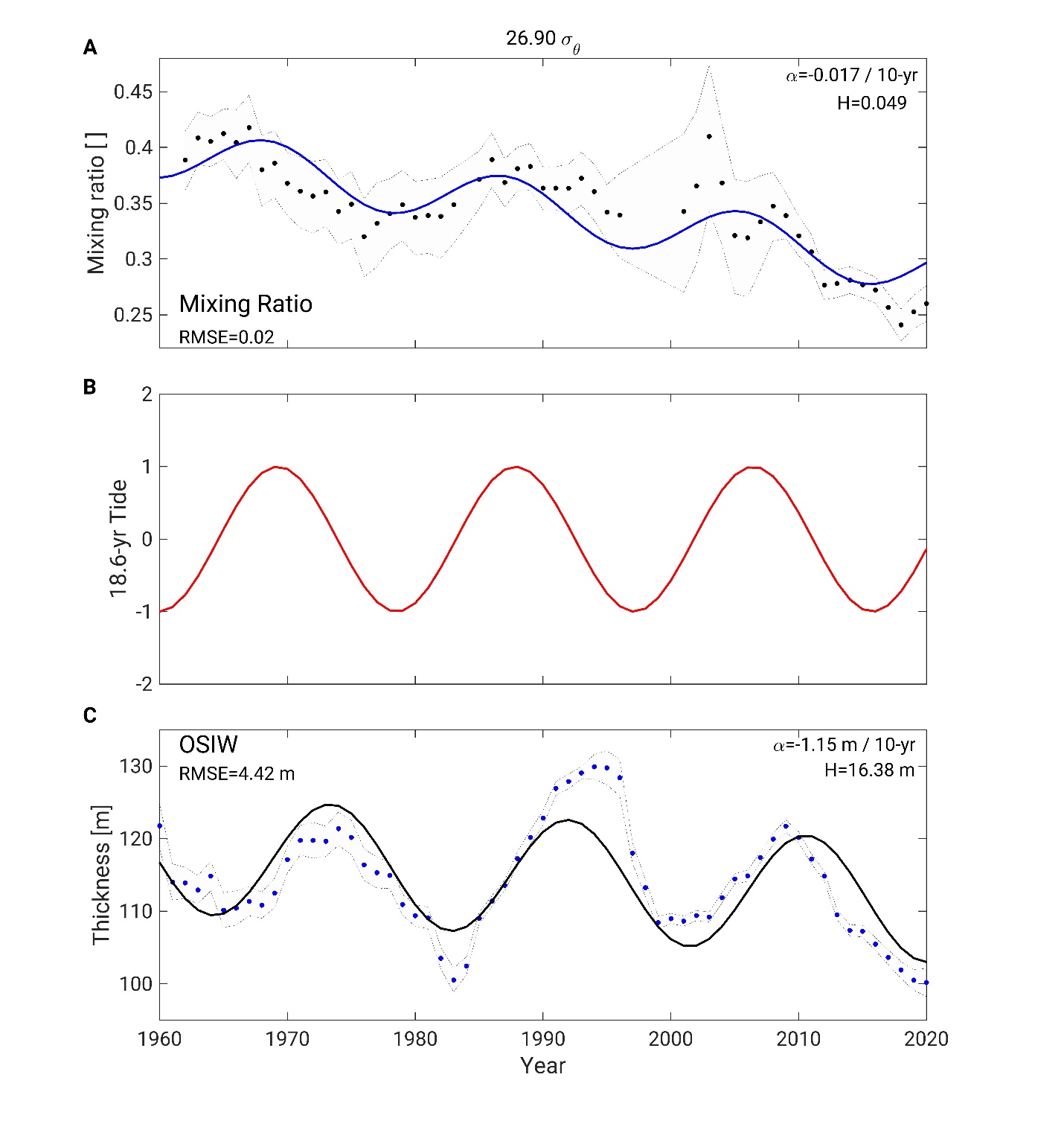


**Fig. S5. Time series of observed and predicted yearly properties in the Sea of Okhotsk and western subarctic Pacific at 26.9 σ_θ_.** Dots represents (mapped) observations and line predict data for (A) mixing ratio of OSIW in OYW, (C) OSIW layer thickness at 26.9 σ_θ_, and (B) the 18.6-yr diurnal tidal cycle. The mixing ratio time series is a composite of the winter (Fig. 8D), summer (Fig. S3) and fall (Fig. S4) time series.

**
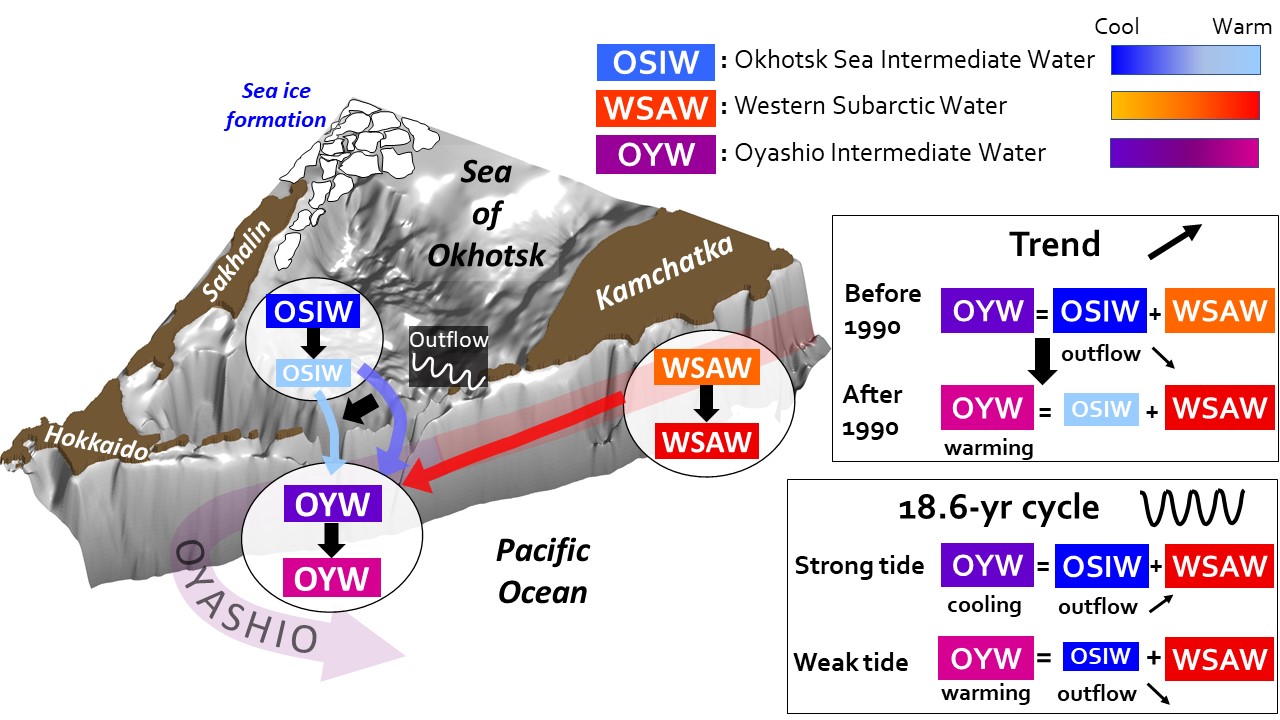
**

**Fig. S6.** **Schematic of the formation of Oyashio Intermediate Water, its changes and future forecast.** The Oyashio Intermediate Water is formed by mixing of the Western Subarctic Water and the Okhotsk Sea Intermediate Water. The properties (temperature, salinity, iron content, etc.) of Oyashio Intermediate Water vary as the mixing ratio between Western Subarctic Water and Okhotsk Sea Intermediate Water changes.

**Table S1:** **Number of potential temperature/salinity data points for each climatological period at 26.9 σ_θ_.** The numbers between brackets indicate the number of dissolved oxygen data points. For the North Pacific, the numbers indicate data located on the Pacific side of the Kuril Islands between (42°N – 60°N, 142°E – 165°E). Data acquired in 1990 were included in both the 1930-1990 and the 1990-2020 datasets.

|  | Sea of Okhotsk | | | North Pacific | | |
| --- | --- | --- | --- | --- | --- | --- |
|  | Jan-Apr | May-Aug | Sep-Dec | Jan-Apr | May-Aug | Sep-Dec |
| 30-90 | 286 (140) | 3354 (1821) | 1177 (989) | 1491 (663) | 5951 (1592) | 3193 (888) |
| 90-20 | 723 (364) | 1204 (792) | 1350 (628) | 3754 (484) | 4597 (1525) | 5441 (1369) |
| Low tide | 537 (222) | 2259 (1620) | 1169 (790) | 2454 (763) | 4082 (659) | 3786 (1395) |
| High tide | 472 (250) | 2299 (975) | 1358 (794) | 2209 (366) | 5619 (1442) | 3849 (838) |

**Table S2:** **Mixing ratio of OSIW for different seasons and different climatological periods.** The 95% confidence interval is indicated by the number following the ± sign. Bold figures in the Δ and percentage changes rows indicate significant differences between climatological periods.

|  | 26.8-27.0 σ_θ_ | | | 27.1-27.4 σ_θ_ | | |
| --- | --- | --- | --- | --- | --- | --- |
|  | Jan-Apr | May-Aug | Sep-Dec | Jan-Apr | May-Aug | Sep-Dec |
| 30-90 | 0.30 ±0.047 | 0.39 ±0.020 | 0.36 ±0.031 | 0.22 ±0.052 | 0.29 ±0.023 | 0.25 ±0.044 |
| 90-20 | 0.22 ±0.023 | 0.41 ±0.023 | 0.36 ±0.023 | 0.17 ±0.019 | 0.26 ±0.028 | 0.23 ±0.037 |
| Δ Trend | **-0.08** | 0.02 | 0.01 | -0.05 | -0.03 | -0.02 |
| Percentage Change | **-28%** | 4% | 2% | -23% | -10% | -8% |
|  |  |  |  |  |  |  |
| Low tide | 0.24 ±0.026 | 0.40±0.025 | 0.32 ±0.027 | 0.16 ±0.027 | 0.26±0.028 | 0.19 ±0.031 |
| High tide | 0.33 ±0.045 | 0.44 ±0.021 | 0.41 ±0.023 | 0.22 ±0.037 | 0.33 ±0.027 | 0.29 ±0.033 |
| Δ Tide | **0.09** | 0.04 | **0.09** | 0.06 | **0.07** | **0.10** |
| Percentage Change | **38%** | 10% | **28%** | 38% | **27%** | **53%** |

**Table S3:** **Number of potential temperature/salinity data points acquired in weak and strong tidal years for the 1930-1990 and the 1990-2020 datasets at 26.9 σ_θ_.** For the North Pacific, the numbers indicate data located on the Pacific side of the Kuril Islands between (42°N – 60°N, 142°E – 165°E). Bold numbers highlight the number of data used to determine the size of the subsets for the ensemble mapping.

|  | Sea of Okhotsk | | | | | | North Pacific | | | | | |
| --- | --- | --- | --- | --- | --- | --- | --- | --- | --- | --- | --- | --- |
|  | Jan-Apr | | May-Aug | | Sep-Dec | | Jan-Apr | | May-Aug | | Sep-Dec | |
|  | Strong tide years | Weak tide years | Strong tide years | Weak tide years | Strong tide years | Weak tide years | Strong tide years | Weak tide years | Strong tide years | Weak tide years | Strong tide years | Weak tide years |
| 30-90 | 265 | **262** | 2533 | **2479** | 1304 | **724** | 882 | **609** | 3830 | **2121** | 2384 | **809** |
| 90-20 | **358** | 399 | **541** | 789 | **613** | 804 | **1424** | 2330 | **1975** | 2622 | **1936** | 3505 |
